# Patient Narratives as Testimonies to Structural Inequity

**DOI:** 10.1002/hast.70096

**Published:** 2026-10-28

**Authors:** Faith E. Fletcher

**Keywords:** medical education, social determinants of health, patient narratives, structural competency, health equity, bioethics

## Abstract

*As of this year, 2026, medical schools are no longer explicitly required to teach structural competency in order to be accredited by the Liaison Committee on Medical Education. This change marks a significant shift in the priorities that guide medical education in the United States. Structural competency provides a framework for understanding how social, economic, political, and institutional forces shape health outcomes, health care experiences, and opportunities for well‐being. Drawing on research encounters with medically underserved populations, particularly Black women living with HIV, this commentary argues that patient narratives are a powerful tool for helping learners in the health professions understand the lived realities that influence health and health care. As ethics educators, we have a moral obligation to help learners recognize the full humanity of the patients they serve so that these future health professionals are better able to provide respectful, compassionate, holistic, and person‐centered care. In the current political climate, fulfilling this obligation may require moral courage*.

## Perspective

As of 2026, medical schools accredited by the Liaison Committee on Medical Education are no longer explicitly required to teach structural competency. This represents a significant shift in the priorities guiding U.S. medical education. Structural competency is a framework that equips health care professionals to critically examine the social, economic, political, and institutional factors that shape health outcomes. It encourages clinicians to consider how these structural determinants influence patient experiences, access to care, engagement in healthy behaviors, and overall health and well‐being.

As a public health, bioethics, and health equity scholar, I have long integrated structural competency and social determinants of health content into my teachings, drawing on narratives from my research with medically underserved populations, particularly Black women living with HIV. These narratives invite students to move beyond individual‐level explanations for health outcomes. Interviewees’ stories reveal the profound impact of housing instability, poverty, stigma, transportation barriers, and fragmented social support systems on health.

One interview remains particularly vivid in my memory. “Amber's” apartment was in disarray, and the space where I conducted the interview was alarmingly close to, less than ten feet from, a large, aggressive dog that barked throughout our conversation. The dog's training pad was soiled, contributing to an unpleasant odor. About halfway through our discussion came loud, forceful knocking at the door. It was Amber's landlord, delivering an eviction notice. Amber pleaded for an additional day to pay her rent, but the landlord refused. The exchange was tense and hostile. After closing the door, she returned to our conversation visibly embarrassed and emotionally defeated.

Recognizing her distress and my discomfort with the emotionally heightened situation, I offered to resume the interview another day. She insisted on continuing, explaining that she needed the research compensation. In that moment, I was confronted with a stark reminder of the economic realities facing many of the women who participated in my research. The interview was no longer simply about HIV, health care utilization, or treatment adherence. It offered a powerful lens through which to understand the intersection of housing insecurity, financial hardship, chronic stress, and health.

Extensive research engagement with Black women living with HIV has transformed how I think about health professions education. A brief clinical encounter may not reveal the eviction notice delivered earlier that day, the difficult choice between paying rent and buying food, or the weightiness of navigating social, economic, and medical challenges. However, establishing rapport and creating space for patients to engage in critical dialogue can reveal important aspects of their lives that influence health and health care experiences. Without understanding these broader circumstances, clinicians may attribute poor health outcomes to personal choices or individual failings rather than recognizing the structural barriers that constrain options and shape behavior.

Narrative‐based approaches to education create opportunities for ethical reflection and encourage students to consider questions about justice, power, responsibility, and the role of health care institutions in addressing social inequities. Rather than viewing health inequities as inevitable and fixed for certain groups of people, learners are challenged to ask why inequities exist and what actions clinicians, health systems, researchers, and policymakers can take to address them. Although some clinicians may not view social inequities as part of their professional responsibilities, understanding how these inequities impact patients is essential to delivering whole‐person care.

The next generation of health professionals need to be taught more than clinical knowledge. As an ethics educator, I use narratives to emphasize that HIV medication nonadherence may reflect barriers such as stigma and housing instability rather than a patient's unwillingness to adhere to treatment. Attention to such barriers can help clinicians develop more effective, compassionate, and patient‐centered approaches to care. Those educating future health professionals have a moral responsibility to help learners recognize the full humanity of their patients. In the current political climate, fulfilling this obligation may require considerable moral courage.

*Views expressed in this commentary are those of the author and do not necessarily reflect those of Baylor College of Medicine*.

